# Association of Health Literacy with Sociodemographic Factors and Medication Adherence Among Primary Health Care Users in Montenegro

**DOI:** 10.3390/healthcare14030374

**Published:** 2026-02-02

**Authors:** Amela Rastoder Celebic, Snezana Radovanovic, Ivana Simic Vukomanovic, Milos Stepovic, Jovana Radovanovic Selakovic, Viktor Selakovic, Olgica Mihaljevic, Katarina Janicijevic, Svetlana Radevic, Sanja Ilic, Marija Sorak, Nela Djonovic, Batric Babovic, Stefan Milojevic, Mihael Djacic, Radica Zivkovic Zaric

**Affiliations:** 1 Faculty of Medical Sciences, University of Kragujevac, 34000 Kragujevac, Serbia; amelarastodercelebic@gmail.com (A.R.C.); sanjailic84@yahoo.com (S.I.); stefan.milojevic@fmn.kg.ac.rs (S.M.); mihaelsanja@yahoo.com (M.D.); 2 Department of Social Medicine, Faculty of Medical Sciences, University of Kragujevac, 34000 Kragujevac, Serbia; jovanarad@yahoo.com (S.R.); drivanasimic@gmail.com (I.S.V.); kaja.andreja@yahoo.com (K.J.); cecaradevic@yahoo.com (S.R.); 3 Institute of Public Health Kragujevac, 34000 Kragujevac, Serbia; ndjonovic@fmn.kg.ac.rs; 4 Department of Anatomy, Faculty of Medical Sciences, University of Kragujevac, 34000 Kragujevac, Serbia; stepovicmilos@yahoo.com; 5 Department of Epidemiology, Faculty of Medical Sciences, University of Kragujevac, 34000 Kragujevac, Serbia; radovanovicjovanaaa@gmail.com; 6 Department of Communication Skills, Ethics, and Psychology, Faculty of Medical Sciences, University of Kragujevac, 34000 Kragujevac, Serbia; selakovicviktor@gmail.com; 7 Department of Pathophysiology, Faculty of Medical Sciences, University of Kragujevac, 34000 Kragujevac, Serbia; vrndic07@yahoo.com; 8 Department of Gynecology and Obstetrics, Faculty of Medical Sciences, University of Kragujevac, 34000 Kragujevac, Serbia; 9 Department of Hygiene and Ecology, Faculty of Medical Sciences, University of Kragujevac, 34000 Kragujevac, Serbia; 10 Department of Nephrology, Clinical Center of Montenegro, 81000 Podgorica, Montenegro; babovicbatric@gmail.com; 11 Faculty of Medicine, University of Montenegro, 81000 Podgorica, Montenegro; 12 Faculty of Business Economics, EDUCONS University, 21208 Sremska Kamenica, Serbia; 13 Special Psychiatric Hospital “Dr Slavoljub Bakalović”, 26300 Vrsac, Serbia; 14 Department of Pharmacology and Toxicology, Faculty of Medical Sciences, University of Kragujevac, 34000 Kragujevac, Serbia; radica_zivkovic@yahoo.com

**Keywords:** health literacy, medication adherence, primary care, socioeconomic factors, Montenegro

## Abstract

Background/Objectives: Health literacy represents the ability to access, understand, appraise, and apply health information for making appropriate health decisions. It is closely linked to education, income, employment, and overall health outcomes. Limited health literacy is associated with poor self-care, inadequate treatment adherence, and increased healthcare utilization. This study aimed to assess the level of health and medication adherence behaviors among primary health care users in Montenegro and examine its association with key demographic and socioeconomic factors. Methods: A cross-sectional, multicenter study was conducted among 202 primary health care users at the Primary Healthcare Center Danilovgrad, Plav and Ulcinj, Montenegro. Data were collected using a demographic questionnaire, the standardized European Health Literacy Questionnaire (HLS-EU-Q-47), and the Attitudes towards Medication Adherence Self-Reported Questionnaire (ADHERE-7). Statistical analyses included descriptive statistics, χ^2^ tests, and univariate and multivariate regression. Results: The mean HLS-EU-Q Index was 33.55 ± 8.05. Significant differences in literacy levels were observed by age (*p* = 0.022), material status (*p* = 0.043), and self-rated health (*p* = 0.020). In multivariate ordinal regression analysis, lower income (<400 €) was associated with lower odds of belonging to a higher health literacy category (OR = 0.22, 95% CI: 0.02–0.92, *p* = 0.039), while no statistically significant associations were observed for gender, education level, or employment status after adjustment. The mean ADHERE-7 score of the study population was 21.78 ± 5.19. When analyzed in relation to the level of health literacy, the highest mean ADHERE-7 score was observed among participants with excellent health literacy (24.28 ± 4.90). Lower levels of health literacy were associated with lower odds of belonging to higher health literacy categories among participants reporting selected non-adherence behaviors, including missing therapy 3–4 times per week (OR = 0.30), frequently skipping prescribed medication when feeling well (OR = 0.03), and reducing or omitting therapy due to perceived lack of benefit or high costs (OR range: 0.10–0.31). Conclusions: Health literacy among primary care users in Montenegro is moderate, with a substantial proportion exhibiting limited literacy. Low income is a key determinant of limited literacy, and limited health literacy was associated with poorer medication adherence. Targeted educational and policy interventions are needed to improve health literacy and reduce health inequalities.

## 1. Introduction

The World Health Organization defines health literacy as the cognitive and social skills that determine the motivation and ability of individuals to gain access to, understand, and use information in ways that promote and maintain good health. It can also be defined as the ability of citizens to make sound health-related decisions in everyday life [1,2].

Health literacy is one of the key pillars of health promotion and disease prevention. It encompasses knowledge, motivation, and competence to access, understand, appraise, and apply health information necessary for decision-making regarding health care and maintaining good health and quality of life [3].

Inadequate health literacy is more common among the elderly (especially those over 65), individuals with low education levels, poor health, multiple chronic conditions, the poor, certain racial and ethnic minorities, refugees, and immigrants [4,5]. Health literacy level is linked to patient–physician communication, treatment outcomes, and the quality of health care. Health care is a shared responsibility between patients and physicians; they must communicate clearly to make responsible health-related decisions [6,7].

Family physicians face numerous problems caused by inadequate health literacy daily, including improper medication use, misunderstanding of treatment advice, misperception of health status, infrequent preventive checkups, frequent medical visits, poor self-care knowledge, communication difficulties, incomplete medical histories, and missed appointments or diagnostic procedures [8,9,10].

In addition to inadequate health literacy, other factors may further influence patient behavior and healthcare utilization. These include distrust in medical professionals, particularly in healthcare systems with limited resources, cognitive impairments related to aging or chronic disease, and reliance on inadequate or unreliable sources of health information, such as non-verified internet content or informal advice networks [6,7]. Such factors may exacerbate misunderstandings of medical advice, reduce adherence to treatment, and contribute to suboptimal health outcomes.

Previous studies have shown that low socioeconomic status is a major risk factor for poor health literacy. Deprivation, poverty, and social inequality are strongly associated with inadequate health literacy. People with lower socioeconomic status (unemployment, low income) tend to have poorer health, are more prone to chronic diseases and injuries, use fewer preventive services, have worse mental health, and face higher mortality rates [11].

The prevalence of inadequate health literacy ranges between 32% and 59%. Differences are observed according to gender, age, marital status, type of settlement, education level, employment status, material status, self-rated health, frequency of visits to family physicians, hospitalizations, number of diseases, and health-related lifestyle choices [12].

Health literacy level can also influence risk perception, disease perception, and health behaviors. This suggests that inadequate health literacy is common among vulnerable population groups and should be recognized by family physicians caring for such patients. Physicians must address literacy-related challenges among patients with multiple diseases, those who underuse preventive and health promotion services, and those who are non-adherent to treatment [13].

In Montenegro, public health education and information dissemination are primarily addressed through national public health policies and health promotion strategies coordinated by the Ministry of Health and the Institute of Public Health. These initiatives focus on disease prevention, promotion of healthy lifestyles, and improving access to health information through primary healthcare services. However, structured and systematically evaluated health literacy-specific programs remain limited, highlighting the need for empirical data to support the development of targeted interventions and evidence-based health literacy policies.

The aim of this study was to assess the level of health literacy among primary health care users in Montenegro and to examine its association with key demographic, socioeconomic, and health-related factors. Specifically, the study evaluated the prevalence of limited health literacy, examined its associations with income, material status, and self-rated health using univariate and multivariate regression, and explored the relationship between health literacy and medication adherence, including specific non-adherence behaviors measured by the ADHERE-7 scale. The findings are intended to inform public health strategies and support the integration of health literacy improvement measures into primary care and health policy planning in Montenegro.

## 2. Materials and Methods

The study was conducted as a cross-sectional, multicenter study on a sample of 202 users of primary health care in Montenegro between 1 November and 15 December 2025. Participants were recruited from primary healthcare centers in Danilovgrad, Plav, and Ulcinj. The statistical power of the study was determined using the G*Power 3.1.2 program (Heinrich Heine University Düsseldorf, Düsseldorf, Germany). Based on data from a study with a similar design [13], and using the χ^2^ test, with a type I error probability of α = 0.05 and a power of 0.95, the required sample size was calculated to be 193 respondents, rounded to 200 participants.

Ethical approvals for conducting the study were obtained from three primary healthcare institutions in Montenegro. The Primary Healthcare Center Danilovgrad issued approval under the reference number 01/25-2139, the Primary Healthcare Center Plav under the reference number 332A, and the Primary Healthcare Center Ulcinj under the reference number 2004.

Inclusion criteria: participants of both sexes, users of primary health care, aged 19 years or older, able to understand the nature of the study, and who signed an informed consent form. Exclusion criteria: individuals younger than 19 years and those unable to understand the study.

As a research instrument, in addition to a general questionnaire on demographic and socioeconomic characteristics, the standardized European Health Literacy Questionnaire (HLS-EU-Q-47) was used to assess health literacy. This instrument consists of 47 items addressing access, understanding, appraisal, and application of health-related information across three domains: disease prevention, health care, and health promotion. For each item, respondents rated the perceived difficulty of the described task or situation on a four-point Likert scale (1 = very difficult, 2 = difficult, 3 = easy, and 4 = very easy), with a possible minimum mean score of 1 and a maximum mean score of 4. For respondents who answered at least 80% of all items, a comprehensive general Health Literacy Index (HLS-EU-Q Index) was calculated using the formula:

Index = (mean − 1) × (50/3)

The resulting scores range from 0 to 50, where 0 represents the lowest possible and 50 the highest possible level of health literacy. Based on the obtained index values, participants were classified into four categories of health literacy levels: Inadequate (0–25); Problematic (>25–33); Sufficient (>33–42); Excellent (>42–50). For identifying vulnerable groups, the “inadequate” and “problematic” levels were combined into a single limited health literacy category (0–33) [3,13].

Medication adherence was assessed was assessed using the Attitudes towards Medication Adherence Self-Reported Questionnaire (ADHERE-7), a validated self-administered instrument designed to evaluate patients’ attitudes and behaviors related to medication adherence. The questionnaire consists of seven items addressing the regularity of medication use, missed doses, discontinuation of therapy, and the ability to manage prescribed treatment in everyday life. Responses are scored using a Likert-type scale, with higher total scores indicating better adherence. In the original validation study, the internal consistency of the ADHERE-7 was evaluated using Cronbach’s alpha, demonstrating acceptable to good reliability, with reported values of α = 0.617 for the aversion non-adherence factor and α = 0.714 for the comfort non-adherence factor, supporting its reliability for assessing different dimensions of medication adherence behavior [14].

In statistical data processing, continuous variables were presented as mean ± standard deviation, and categorical variables as proportions of respondents with specific outcomes. Due to non-normal distribution of several variables and unequal group sizes across age categories, non-parametric statistical tests were applied where appropriate. The χ^2^ test was used to compare differences in the frequency of categorical variables. Ordinal logistic regression was used to examine associations between independent variables and health literacy level. The outcome variable was health literacy, ordered from limited to excellent, with “excellent health literacy” set as the reference category. Odds ratios (ORs) below 1 therefore indicate lower odds of belonging to a higher health literacy category. The proportional odds (parallel lines) assumption was formally tested. As this assumption was not fully met in the final models, the estimated odds ratios should be interpreted as average associations across outcome thresholds rather than causal effects. All results with a *p*-value < 0.05 were considered statistically significant. Statistical analyses were performed using the Statistical Package for the Social Sciences (SPSS), version 20.0 (SPSS Inc., Chicago, IL, USA).

## 3. Results

The study included 202 participants, evenly distributed by sex (50% male, 50% female) (χ^2^ = 3.446, *p* = 0.632). Most respondents were middle-aged or older and predominantly from urban areas. The majority were married or in a partnership and had completed secondary education, while nearly one-third had higher or university-level education. More than half were employed, whereas about two-fifths were unemployed, retired, or unable to work. Most participants reported average material status and a monthly income between 200 and 600 euros. Regarding self-rated health, the largest proportion of respondents described their health as average or good (Table 1).

The mean HLS-EU-Q Index value was 33.55 ± 8.05 (range: 10.28–49.42). Across health literacy categories, the mean index values were: Inadequate literacy: 19.18 ± 4.12, Problematic literacy: 29.29 ± 2.20, Sufficient literacy: 37.21 ± 2.19, Excellent literacy: 44.95 ± 2.23 (Kruskal–Wallis test, *p* < 0.001). By category, 32 participants (15.8%) had inadequate health literacy, 44 (21.7%) problematic literacy, 103 (51.1%) sufficient literacy, and 23 (11.4%) excellent literacy.

Analysis of health literacy in relation to demographic and socioeconomic characteristics revealed statistically significant differences according to age (χ^2^ = 20.829, *p* = 0.022), material status (χ^2^ = 15.946, *p* = 0.043), and health status (χ^2^ = 18.198, *p* = 0.020). The highest percentage of participants with both limited (32.9%) and excellent (30.4%) health literacy was in the 45–54 years age group, while the largest proportion of participants with sufficient literacy (34%) was in the 65+ age group. The lowest percentage of participants with limited or inadequate literacy (2.6%) was among those aged 19–24.

Regarding material status, the majority of participants in each literacy category had average material status: 44.7% in the limited literacy group, 47.6% in the sufficient group, and 52.2% in the excellent group. Statistical analysis showed that very good material status was exclusively associated with sufficient literacy (9.7%), while none of the participants with very poor material status demonstrated excellent literacy. More than one-third of participants (34.8%) with good health status demonstrated excellent health literacy, while 36.9% of those with average health status had limited literacy (Table 2).

To facilitate the regression analysis, we grouped several categorical variables into fewer categories. Age was regrouped into three categories (19–34, 35–54, 55+), Settlement into three (Urban, Suburban, Rural), Marital status into three (Married, Widowed, Divorced), Education into three (Primary or lower, Secondary, Higher/university), Employment into two (Employed, Unemployed), Income into three (<400 €, 400–800 €, >800 €), Material status into three (Poor, Average, Good), and Health self-assessment into three (Poor, Average, Good). This regrouping was performed to ensure adequate sample size per category, avoid sparse data, and maintain stability and validity of the ordinal regression estimates. The final ordinal regression model demonstrated acceptable fit and modest explanatory power (−2 Log Likelihood decreased from 307.58 to 282.79, Chi-Square = 24.79, df = 16, *p* = 0.074; pseudo R^2^: Cox & Snell = 0.130, Nagelkerke = 0.153, McFadden = 0.073; Pearson χ^2^ = 291.74, *p* = 0.086; Deviance χ^2^ = 261.60, *p* = 0.461; parallel lines test χ^2^ = 29.63, df = 16, *p* = 0.020).

In univariate models, several variables showed significant associations with health literacy levels. Participants living in urban areas were more likely to have higher health literacy compared to those in rural areas (OR = 2.21, 95% CI: 1.10–4.45, *p* = 0.025), while those living in suburban areas showed a non-significant trend toward higher literacy (OR = 1.48, 95% CI: 0.73–3.04, *p* = 0.279). Low income (<400 €) was significantly associated with lower health literacy (OR = 0.27, 95% CI: 0.08–0.93, *p* = 0.039), whereas moderate income (400–800 €) was not significant (OR = 0.45, 95% CI: 0.17–1.08, *p* = 0.161).

Regarding material status, participants reporting “Poor” material conditions had lower odds of higher health literacy compared to those with “Good” status (OR = 0.35, 95% CI: 0.16–0.79, *p* = 0.010), and those with “Average” material status also had a reduced likelihood (OR = 0.48, 95% CI: 0.23–0.99, *p* = 0.049). For self-assessed health, participants with “Poor” health had lower odds of higher health literacy (OR = 0.37, 95% CI: 0.18–0.76, *p* = 0.007), and those with “Average” health similarly showed reduced odds (OR = 0.33, 95% CI: 0.15–0.66, *p* = 0.002). Other variables, including gender, age, marital status, education, and employment, did not reach statistical significance in univariate models.

After adjusting for all predictors simultaneously in the multivariate model, only low income (<400 €) remained significantly associated with lower health literacy (OR = 0.22, 95% CI: 0.02–0.92, *p* = 0.039). Other predictors, including urban or suburban settlement, gender, age, marital status, education, employment, material status, and self-assessed health, lost statistical significance after adjustment, indicating that their apparent associations in univariate analyses were confounded by other variables (Table 3).

**Figure 1:**
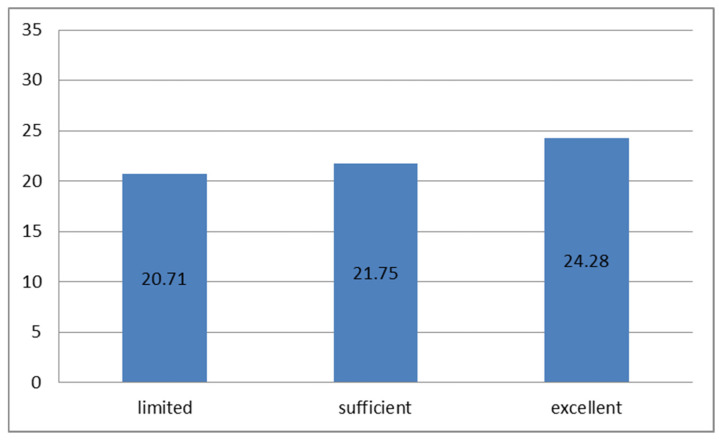
The mean ADHERE-7 score.

The mean ADHERE-7 score in the study population was 21.78 ± 5.19 (minimum 8, maximum 28). With respect to the level of health literacy, the mean ADHERE-7 score was 20.71 ± 4.98 among participants with limited health literacy, 21.75 ± 5.26 among those with adequate health literacy, and 24.28 ± 4.90 among participants with excellent health literacy. These findings indicate that individuals with excellent health literacy had a statistically significantly higher ADHERE-7 score (independent samples Kruskal–Wallis test, *p* = 0.009), as shown in Figure 1.

With regard to sociodemographic characteristics, analysis of ADHERE-7 scores showed that the mean ADHERE-7 score decreased with increasing age and was significantly higher among participants living in urban areas. It should be noted that the youngest age group (19–24 years) included a small number of participants (n = 10), which may have contributed to unstable estimates of health literacy and ADHERE-7 scores in this subgroup. Widowed participants had the lowest mean ADHERE-7 scores compared with other marital status categories. In addition, the level of education was positively correlated with ADHERE-7 scores, with the highest mean values observed among participants with higher and university-level education. Significant differences in ADHERE-7 scores were also identified for other independent variables, including employment status (the lowest mean scores among participants unable to work), income (higher mean scores among participants with higher monthly income), material status (higher index values corresponding to better material status and vice versa), and health status (the highest mean scores among participants reporting very good health status), as presented in Table 4.

Table 5 presents the association between health literacy level and medication adherence behaviors. Lower levels of health literacy were associated with lower odds of reporting optimal adherence behaviors. These findings reflect statistical associations and should not be interpreted as evidence of a causal relationship.

The final ordinal regression model including all predictors demonstrated acceptable fit and modest explanatory power (−2 Log Likelihood decreased from 264.75 to 223.45, Chi-Square = 41.30, df = 22, *p* = 0.008; Nagelkerke R^2^ = 0.240; Pearson χ^2^ = 206.37, *p* = 0.402; Deviance χ^2^ = 199.38, *p* = 0.539; test of parallel lines *p* = 0.029).

In univariate models, several behavioral factors were significantly associated with lower health literacy. Missing 3–4 doses in the past week (Q1) was associated with a lower likelihood of higher health literacy (OR = 0.23, 95% CI: 0.07–0.75, *p* = 0.015), and missing ≥5 doses in the past week showed a trend toward lower literacy (OR = 0.18, 95% CI: 0.03–1.06, *p* = 0.056). Skipping doses when feeling well (Q2) ≥ 5 times was significantly associated with lower health literacy (OR = 0.03, 95% CI: 0.00–0.24, *p* = 0.001). Reducing or not taking medication because it was believed to be ineffective (Q5) was significantly associated with lower literacy across all categories (1–2 times: OR = 0.32, 95% CI: 0.14–0.75, *p* = 0.009; 3–4 times: OR = 0.25, 95% CI: 0.11–0.55, *p* = 0.004; ≥5 times: OR = 0.10, 95% CI: 0.03–0.36, *p* = 0.003). Skipping doses due to high costs (Q6) 1–2 times or ≥5 times also significantly decreased the odds of higher health literacy (OR = 0.28, 95% CI: 0.09–0.85, *p* = 0.025; OR = 0.13, 95% CI: 0.02–0.85, *p* = 0.034, respectively). Other predictors, including forgetting doses in the past month (Q3), skipping doses because the medicine was harmful (Q4), and difficulty managing therapy (Q7), did not reach statistical significance in the univariate models, although trends were observed.

When all predictors were included simultaneously in the multivariate ordinal regression model, several associations remained statistically significant. Missing 3–4 doses in the past week (Q1) remained significant (OR = 0.30, 95% CI: 0.11–0.82, *p* = 0.020). Skipping doses because the medication was believed to be ineffective (Q5) continued to be significant across all categories (1–2 times: OR = 0.31, 95% CI: 0.13–0.73, *p* = 0.009; 3–4 times: OR = 0.25, 95% CI: 0.11–0.55, *p* = 0.004; ≥5 times: OR = 0.10, 95% CI: 0.03–0.36, *p* = 0.003). Skipping doses when feeling well (Q2) ≥ 5 times (OR = 0.03, 95% CI: 0.01–0.25, *p* = 0.015) and skipping doses due to high costs (Q6) 1–2 times and ≥5 times also remained significant (OR = 0.28, 95% CI: 0.09–0.85, *p* = 0.031; OR = 0.13, 95% CI: 0.02–0.85, *p* = 0.031, respectively). Other predictors, including Q2, Q3, Q4, and Q7, lost statistical significance in the multivariate model (Table 5).

## 4. Discussion

The present study assessed health literacy among primary healthcare users in, Montenegro, and evaluated its association with demographic and socioeconomic factors. Given the cross-sectional design and the violation of the proportional odds assumption, the observed relationships should be interpreted as associations rather than causal effects.

The findings indicate that the average health literacy level in this population is moderate, with a considerable proportion of participants exhibiting inadequate or problematic health literacy. These results are consistent with prior studies conducted in similar populations across Europe and the Balkan region, highlighting that health literacy is significantly influenced by social determinants of health, particularly income [15,16,17,18,19].

Similar levels of limited or problematic health literacy have been reported in studies from several European and Balkan countries. Research conducted in Croatia, Serbia, and Slovenia has shown that between one-third and one-half of adult primary care users exhibit limited health literacy, particularly among older adults, individuals with lower education, and those with lower socioeconomic status [20,21,22]. Studies from Western European countries have also demonstrated social gradients in health literacy, although the prevalence of limited literacy tends to be lower in countries with more developed health education systems [23]. The findings from Montenegro therefore align with regional patterns while also underscoring persistent socioeconomic inequalities in health literacy across Europe.

Our analysis showed that several sociodemographic factors, such residence type, income, material status and health assessment were associated with lower health literacy in univariate analyses. However, in multivariate models, only low income (<400 €) remained significantly associated with limited health literacy. These findings align with existing literature, which consistently demonstrates that socioeconomic disadvantages are closely linked with limited health literacy. Individuals with lower education may have less capacity to access, comprehend, and apply health information, which may be associated with poorer health outcomes [24]. Similarly, lower income often restricts access to healthcare resources and educational opportunities, compounding the effects of limited health literacy [25].

Interestingly, participants originating from urban areas demonstrated better health literacy compared to those from rural areas [26]. This may reflect greater availability of healthcare services, educational resources, and health promotion initiatives in urban settings. Access to accurate health information and opportunities for patient education are more readily available in urban environments, which can enhance individuals’ ability to navigate healthcare systems and adopt preventive health behaviors [27].

Age was also associated with health literacy level. The largest proportion of participants with both limited (32.9%) and excellent (30.4%) health literacy belonged to the 45–54 years age group, indicating considerable heterogeneity within this middle-aged population. Conversely, the lowest proportion of participants with limited health literacy (2.6%) was observed among the youngest group, aged 19–24 years. The extremely high health literacy and adherence observed in the youngest age group should be interpreted with caution. This subgroup included a limited number of participants, which may have resulted in inflated mean values and an apparently monotonic trend across age groups. Such age-group imbalance may reflect selection bias and limits the generalizability of age-specific comparisons. But, also, this finding may reflect the influence of modern educational curricula and digital literacy, as younger adults are more accustomed to accessing information online and may have better general literacy skills [28]. At the same time, it highlights the need for targeted interventions for middle-aged populations who may face cumulative challenges, such as multiple chronic conditions, limited time for self-care, or greater exposure to complex health information without adequate support [29].

Across all literacy categories, the majority of participants reported average material status. However, very good material status was exclusively associated with sufficient health literacy (9.7%), whereas participants with very poor material status did not demonstrate excellent literacy. Individuals with limited financial resources may face barriers in accessing health services, acquiring health-related information, and adopting healthy lifestyles, which contributes to a cycle of vulnerability [30].

The study also highlighted the relationship between health literacy and self-reported health status in univariate model. More than one-third of participants with good health status (34.8%) demonstrated excellent health literacy, whereas 36.9% of those with average health status exhibited limited literacy. This association supports previous findings suggesting that higher health literacy enables individuals to better manage their health, adhere to preventive measures, and engage in health-promoting behaviors [31]. Conversely, inadequate health literacy may be associated with less effective self-management and poorer health outcomes.

Our findings have important implications for public health policy and primary care practice like targeted educational interventions aimed at vulnerable population [32,33]. Such interventions may include tailored health communication, simplified educational materials, and community-based programs to enhance health knowledge and self-management skills. Second, the urban–rural disparity observed in health literacy emphasizes the necessity to improve access to health information and healthcare services in rural communities. Strategies could include mobile health initiatives, outreach programs, and the use of digital platforms to bridge gaps in health literacy and reduce inequities. Third, the relationship between age and literacy suggests that interventions should be adapted to specific life stages, addressing the particular needs and challenges of middle-aged adults while reinforcing skills among younger populations.

Strengthening health communication skills among primary care physicians through training in plain language and teach-back methods may further enhance patient understanding. In addition, the integration of health literacy principles into national health promotion campaigns, school curricula, and community-based interventions could contribute to long-term improvements. Digital health tools and mobile health applications may also represent valuable resources for improving access to reliable health information, especially in rural and underserved areas [33,34].

Moreover, the study underscores the importance of integrating health literacy assessment into routine primary care practice. Family physicians and primary care providers play a critical role in identifying patients with limited health literacy and providing appropriate support [35]. This may involve using plain language communication, verifying patient understanding through teach-back methods, and involving caregivers when necessary. By recognizing and addressing health literacy gaps, healthcare providers can enhance patient engagement, adherence to treatment, and overall health outcomes.

Participants with limited health literacy missed prescribed therapy more frequently, omitted medication intake, and experienced greater difficulty managing their therapy, whereas those with excellent health literacy had significantly higher ADHERE-7 scores. Medication adherence was examined as a behavioral outcome influenced by health literacy, which reflects patients’ capacity to obtain and apply health information in everyday therapeutic decision-making. These findings are consistent with recent evidence demonstrating that low health literacy is associated with poorer medication adherence, particularly in chronic disease populations. A systematic review of polypharmacy patients revealed that individuals with low health literacy had higher rates of unintentional non-adherence and more frequent prescription misinterpretations, and highlighted that pharmacist-led education and visual tools can substantially improve adherence outcomes [36].

Our results also showed that higher education, urban residence, and better self-reported health were associated with better adherence and higher ADHERE-7 scores, aligning with findings from hypertension research where health literacy significantly predicted medication adherence and explained a notable portion of adherence variance, especially among individuals with higher education and income [37]. This supports the concept that health literacy enhances a patient’s ability to understand complex regimens and engage in consistent medication use. Notably, regression analysis in our study confirmed that even after controlling for other factors, limited health literacy remained associated with omitted medication use.

A review of chronic disease populations reported that many studies found positive relationships between health literacy and medication adherence, though some reported mixed or no associations, underscoring the complexity of adherence determinants and the influence of individual, social, and cultural contexts [38]. Another synthesis of research specifically among older adults indicated that the majority of studies documented positive associations between self-reported health literacy and medication adherence, despite variability in measures used across studies [39]. These patterns mirror our findings, which showed a clear gradient between literacy levels and adherence outcomes, particularly in older, less educated, or socioeconomically disadvantaged groups.

Beyond observational associations, interventional studies show that improving health literacy can enhance adherence. For example, mobile health (M-health) literacy programs significantly increased health literacy scores and medication adherence among hypertensive patients, indicating that targeted literacy interventions may improve clinical outcomes [37,40]. This evidence suggests that health literacy enhancement strategies may be associated with improved adherence in interventional settings.

Future research may also consider conducting similar studies in community pharmacy settings. Community pharmacies represent highly accessible points of contact within the healthcare system and play an important role in patient education and medication counseling. Assessing health literacy and medication adherence behavior in this context could further support public awareness initiatives and contribute to improved medication adherence through pharmacist-led interventions.

While the study provides valuable insights into health literacy and medication adherence behavior in Montenegro, certain limitations should be acknowledged. The small and uneven sample size across age groups, particularly in the youngest category, may have influenced the observed trends and should be considered when interpreting the results. Additionally, the proportional odds assumption was not fully satisfied in the ordinal regression models, which may limit the precision of the estimated odds ratios across outcome categories. The cross-sectional design limits causal inference between health literacy and medication adherence, and the sample, although representative of primary care users, may not reflect the broader population of Montenegro. Self-reported measures, including income, material status, and health status, may be subject to reporting bias. Nevertheless, the use of the validated HLS-EU-Q-47 instrument, as well as ADHERE-7, strengthens the reliability of the findings and allows for comparison with other European populations.

## 5. Conclusions

In conclusion, health literacy among primary healthcare users in Montenegro demonstrates that low income emerged as the variable with the strongest association with limited literacy. While other sociodemographic factors showed associations in univariate analyses, these were not statistically significant after adjustment, highlighting the central role of economic resources.

Limited health literacy is also consistently associated with poorer medication adherence, including missed, skipped or reduced doses when patients felt well or believed that medication was not helpful, as well as with the influence of cost of medication, suggesting its relationship with pharmacotherapeutic behaviors.

These findings emphasize the need for targeted public health interventions focusing on economically disadvantaged populations to improve both health literacy and medication adherence. Strategies may include simplified educational materials, financial support programs, and community- or technology-based interventions to facilitate access to health information. Overall, health literacy should be considered a core component of public health strategies, integrated into clinical practice, and addressed through comprehensive educational and policy interventions to reduce health inequities in Montenegro. Higher health literacy is associated with better health decision-making, self-care, and medication adherence, suggesting that health literacy may represent an important target for public health interventions. These results additionally suggest that health literacy should be considered a key component of public health strategies, integrated into clinical practice, and addressed through comprehensive educational and policy interventions to reduce health inequities in Montenegro.

## Figures and Tables

**Table 1:** Demographic and Socioeconomic Characteristics of the Study Population.

Variable	Category	N (%)
Gender	Male	101 (50%)
	Female	101 (50%)
Age group	19–24	10 (5%)
	25–34	16 (7.9%)
	35–44	25 (12.4%)
	45–54	52 (25.7%)
	55–64	41 (20.3%)
	65+	58 (28.7%)
Place of residence	Urban area	97 (48%)
	Suburban area	54 (26.7%)
	Rural area	51 (25.2%)
Marital status	Married/in partnership	135 (66.8%)
	Widowed	29 (14.4%)
	Divorced	38 (18.8%)
Education	Primary or lower	23 (11.4%)
	Secondary	119 (58.9%)
	Higher or university	60 (29.7%)
Employment status	Employed	116 (57.4%)
	Unemployed	12 (5.9%)
	Student/trainee	8 (4%)
	Retired	51 (25.2%)
	Unable to work	5 (2.5%)
	Homemaker	10 (5%)
Monthly income	<200 euros	11 (5.4%)
	200–400 euros	54 (26.7%)
	400–600 euros	57 (28.2%)
	600–800 euros	44 (21.8%)
	>800 euros	36 (17.8%)
Material status	Very poor	11 (5.4%)
	Poor	51 (25.2%)
	Average	95 (47%)
	Good	35 (17.3%)
	Very good	10 (5%)
Self-rated health	Very good	21 (10.4%)
	Good	52 (25.7%)
	Average	71 (35.1%)
	Poor	33 (16.3%)
	Very poor	25 (12.4%)

**Table 2:** Health Literacy in Relation to Demographic and Socioeconomic Characteristics of the Study Population.

		Health Literacy			χ^2^
		Limited N = 76	Sufficient N = 103	Excellent N = 23	
Gender	Male	47 (61.8%)	45 (43.7%)	9 (39.1%)	χ^2^ = 4.561. *p* = 0.102
	Female	29 (38.2%)	58 (56.3%)	14 (60.9%)	
Age groups	19–24	2 (2.6%)	4 (3.9%)	4 (17.4%)	χ^2^ = 20.829. *p* = 0.022 *
	25–34	4 (5.2%)	9 (8.7%)	3 (13.1%)	
	35–44	6 (7.9%)	14 (13.6%)	5 (21.7%)	
	45–54	25 (32.9%)	20 (19.4%)	7 (30.4%)	
	55–64	18 (23.7%)	21 (20.4%)	2 (8.7%)	
	65+	21 (27.7%)	35 (34%)	2 (8.7%)	
Settlement type	Urban area	29 (38.1%)	55 (53.4%)	13 (56.5%)	χ^2^ = 7.564. *p* = 0.109
	Suburban area	19 (25%)	31 (30.1%)	4 (17.4%)	
	Rural area	28 (36.9%)	17 (16.5%)	6 (26.1%)	
Marital status	Married/in partnership	46 (60.5%)	69 (67%)	20 (87%)	χ^2^ = 4.626. *p* = 0.328
	Widowed	10 (13.2%)	16 (15.5%)	3 (13%)	
	Divorced	20 (26.3%)	18 (17.5%)	/	
Education level	Primary or lower	10 (13.2%)	8 (7.7%)	5 (21.7%)	χ^2^ = 9.021. *p* = 0.061
	Secondary	56 (73.6%)	48 (46.6%)	15 (65.3%)	
	Higher or university	10 (13.2%)	47 (45.7%)	3 (13%)	
Employment status	Employed	44 (57.9%)	59 (57.3%)	13 (56.5%)	χ^2^ = 14.039. *p* = 0.171
	Unemployed	4 (5.2%)	8 (7.7%)	0	
	Student/trainee	3 (4%)	3 (2.9%)	2 (8.7%)	
	Retired	18 (23.7%)	29 (28.2%)	4 (17.4%)	
	Unable to work	3 (4%)	0	2 (8.7%)	
	Homemaker	4 (5.2%)	4 (3.9%)	2 (8.7%)	
Income	<200 euros	5 (6.6%)	6 (5.9%)	0	χ^2^ = 12.671. *p* = 0.124
	200–400 euros	28 (36.9%)	21 (20.4%)	5 (21.7%)	
	400–600 euros	19 (25%)	27 (26.2%)	11 (47.9%)	
	600–800 euros	17 (22.3%)	22 (21.3%)	5 (21.7%)	
	>800 euros	7 (9.2%)	27 (26.2%)	2 (8.7%)	
Material status	Very poor	3 (4%)	8 (7.7%)	0	χ^2^ = 15.946. *p* = 0.043 *
	Poor	28 (36.9%)	18 (17.5%)	5 (21.7%)	
	Average	34 (44.7%)	49 (47.6%)	12 (52.2%)	
	Good	11 (14.4%)	18 (17.5%)	6 (26.1%)	
	Very good	0	10 (9.7%)	0	
Self-assessed health	Very good	4 (5.2%)	11 (10.7%)	6 (26.1%)	χ^2^ = 18.198. *p* = 0.020 *
	Good	15 (19.7%)	29 (28.2%)	8 (34.8%)	
	Average	28 (36.9%)	43 (41.7%)	0	
	Poor	16 (21%)	11 (10.7%)	6 (26.1%)	
	Very poor	13 (17.2%)	9 (8.7%)	3 (13%)	

* *p* value less than 0.05 is statistically significant; chi-square test.

**Table 3:** Univariate and Multivariate Regression Analysis of the relationship of Demographic and Socioeconomic Characteristics of the Study Population on Health Literacy.

Predictor	Category	B (Univariate)	B (Multivariate)	Univariate OR (95% CI)	*p*	Multivariate OR (95% CI)	*p*
Gender	Male	−0.513	−0.373	0.60 (0.27–1.02)	0.073	0.69 (0.42–1.21)	0.239
Age	19–34	0.866	0.072	2.37 (0.96–5.22)	0.074	1.08 (0.34–3.35)	0.902
	35–54	0.278	−0.291	1.32 (0.70–2.48)	0.369	0.75 (0.33–1.67)	0.480
Settlement	Urban	0.794	0.428	2.21 (1.10–4.45)	0.025 *	1.53 (0.70–3.10)	0.327
	Suburban	0.392	0.357	1.48 (0.73–3.04)	0.279	1.43 (0.65–3.10)	0.428
Marital status	Married	0.322	0.571	1.38 (0.73–2.61)	0.305	1.77 (0.76–4.12)	0.175
	Widowed	0.322	0.454	1.38 (0.59–3.26)	0.468	1.57 (0.34–7.24)	0.441
Education	Primary or lower	−0.579	0.529	0.56 (0.21–1.51)	0.238	1.70 (0.45–6.08)	0.431
	Secondary	−0.527	0.141	0.59 (0.31–1.14)	0.102	1.15 (0.45–2.91)	0.714
Employment	Employed	−0.314	−0.310	0.73 (0.29–1.51)	0.492	0.73 (0.28–1.88)	0.492
Income	<400 €	−1.313	−1.511	0.27 (0.08–0.93)	0.039 *	0.22 (0.02–0.92)	0.039 *
	400–800 €	−0.800	−0.791	0.45 (0.17–1.08)	0.161	0.45 (0.17–1.08)	0.161
Material status	Poor	−0.094	0.117	0.91 (0.16–1.53)	0.871	0.84 (0.23–3.23)	0.725
	Average	−0.729	−0.171	0.48 (0.23–0.99)	0.049 *	0.84 (0.23–3.23)	0.725
Health self-assessment	Poor	−0.967	−0.917	0.38 (0.12–1.19)	0.096	0.40 (0.14–1.14)	0.105
	Average	−1.104	−0.684	0.33 (0.15–0.66)	0.002 *	0.60 (0.24–1.44)	0.305

Reference categories: Health literacy = Excellent, Gender = Female, Age = 55+, Settlement = Rural, Marital Status = Divorced, Education = Higher/university, Employment = Unemployed, Income = >800 €, Material Status = Good, Health Self-Assessment = Good. * Statistically significant (*p* < 0.05).

**Table 4:** ADHERE-7 score values according to demographic and socio-economic characteristics of the study population.

Variable	Category	ADHERE-7 Score (Mean ± SD)	*p* *
Sex	Male	21.69 ± 4.82	0.812
	Female	21.87 ± 5.57	
Age (years)	19–24	27.33 ± 0.44	<0.001 *
	25–34	26.73 ± 0.86	
	35–44	25.41 ± 0.58	
	45–54	22.30 ± 0.57	
	55–64	20.15 ± 0.76	
	≥65	19.08 ± 0.72	
Place of residence	Urban	23.57 ± 4.58	<0.001 *
	Suburban	20.54 ± 5.24	
	Rural	19.80 ± 5.20	
Marital status	Married	22.75 ± 4.73	<0.001 *
	Widowed	18.39 ± 5.64	
	Divorced	20.97 ± 5.35	
Education level	Primary or lower	16.81 ± 5.09	<0.001 *
	Secondary	22.02 ± 4.79	
	Higher/University	23.23 ± 4.96	
Employment status	Employed	23.41 ± 4.39	<0.001 *
	Unemployed	20.18 ± 3.68	
	Students	22.75 ± 5.80	
	Retired	19.24 ± 5.86	
	Unable to work	17.40 ± 2.50	
	Homemaker	19.22 ± 5.06	
Monthly income	<200€	17.60 ± 5.48	<0.001 *
	200–400€	19.77 ± 4.94	
	400–600€	22.43 ± 5.06	
	600–800€	22.68 ± 4.78	
	>800€	23.83 ± 4.79	
Material status	Very poor	18.10 ± 3.24	<0.001 *
	Poor	18.41 ± 5.49	
	Average	23.16 ± 4.22	
	Good	23.58 ± 5.13	
	Very good	22.70 ± 5.28	
Health status	Very good	27.33 ± 1.65	<0.001 *
	Good	24.28 ± 3.89	
	Average	21.35 ± 4.61	
	Poor	19.15 ± 4.20	
	Very poor	16.34 ± 5.10	

* *p* value less than 0.05 is statistically significant, Independent samples test.

**Table 5:** Univariate and multivariate regression analysis of association between health literacy level and medication adherence behaviors.

Predictor	Category	B (Estimate)	Univariate OR (95% CI)	*p*	Multivariate OR (95% CI)	*p*
How many times have you missed taking your therapy in the past week? (Q1)	1–2 times	1.173	0.37 (0.12–1.13)	0.077	0.36 (0.12–1.13)	0.060
	3–4 times	−0.607	0.23 (0.07–0.75)	0.015 *	0.30 (0.11–0.82)	0.020 *
	≥5 times	−1.009	0.18 (0.03–1.06)	0.056	0.28 (0.05–1.48)	0.135
When I feel well, I skip or reduce taking my prescribed medication (Q2)	1–2 times	−3.677	0.42 (0.11–1.65)	0.211	0.48 (0.15–1.55)	0.221
	3–4 times	−0.725	0.50 (0.14–1.73)	0.277	0.49 (0.14–1.70)	0.256
	≥5 times	−0.835	0.03 (0.00–0.24)	0.001 *	0.03 (0.01–0.25)	0.015 *
How many times in the past month did you forget to take your therapy? (Q3)	1–2 times	1.889	0.78 (0.28–2.18)	0.630	1.12 (0.35–3.56)	0.845
	3–4 times	0.595	1.52 (0.57–4.06)	0.396	1.81 (0.63–5.18)	0.270
	≥5 times	0.601	2.42 (0.90–6.49)	0.079	2.13 (0.79–5.74)	0.137
I skip or reduce a dose because I believe the medicine is harmful (Q4)	1–2 times	0.093	0.92 (0.30–2.86)	0.889	0.91 (0.29–2.87)	0.871
	3–4 times	−2.816	0.76 (0.25–2.27)	0.623	0.76 (0.24–2.25)	0.614
	≥5 times	−2.274	0.62 (0.18–2.11)	0.443	0.63 (0.18–2.18)	0.451
I reduced or did not take a medication because I believed it does not help (Q5)	1–2 times	−2.353	0.32 (0.14–0.75)	0.009 *	0.31 (0.13–0.73)	0.009 *
	3–4 times	−0.205	0.24 (0.11–0.53)	0.001 *	0.25 (0.11–0.55)	0.004 *
	≥5 times	−1.404	0.10 (0.03–0.36)	0.001 *	0.10 (0.03–0.36)	0.003 *
In the past month, due to high costs, have you used your medication less frequently or in smaller amounts? (Q6)	1–2 times	−2.573	0.28 (0.09–0.85)	0.025 *	0.28 (0.09–0.85)	0.031 *
	3–4 times	−0.272	0.74 (0.27–2.01)	0.555	0.73 (0.27–1.99)	0.623
	≥5 times	−0.408	0.13 (0.02–0.85)	0.034 *	0.13 (0.02–0.85)	0.031 *
How difficult is it for you to manage your therapy? (Q7)	Very difficult everyday	−0.485	0.62 (0.22–1.74)	0.360	1.39 (0.26–7.48)	0.703
	Mostly difficult	−1.132	0.32 (0.15–0.72)	0.006 *	0.51 (0.15–1.75)	0.286
	Sometimes difficult	−0.586	0.57 (0.29–1.12)	0.102	0.74 (0.31–1.78)	0.501

Reference categories: “Never missed” for Q1–Q6; “Not difficult at all” for Q7; Health literacy = Excellent. * Statistically significant (*p* < 0.05).

## Data Availability

All data is contained within this article.
